# Early detection of subjective cognitive decline and Alzheimer's disease: Analytical validation of a newly developed pT217‐tau assay

**DOI:** 10.1002/alz.13707

**Published:** 2024-02-08

**Authors:** Hash Brown Taha

**Affiliations:** ^1^ Department of Integrative Biology & Physiology University of California Los Angeles Los Angeles California USA

I read with interest the insightful article by Gonzalez‐Ortiz et al.^1^ regarding the development of a new assay for quantification of phosphorylated threonine 217 tau (pT217‐tau) in the plasma of persons with subjective cognitive decline or early Alzheimer's disease (AD). This novel assay could potentially offer a more precise and earlier diagnosis of persons with AD.

With regard to the analytical validation of the assay, the authors performed experiments relating to within‐ and between‐run[Fig alz13707-fig-0001] stability (Figure S2A, ^1^) dilution linearity (Figure S2B, ^1^) and spike recoveries (Figure S2C, ^1^). The authors showed that the assay provides robust measurements for within‐ and between‐run stability. For dilution linearity, the authors performed such experiments by diluting the plasma samples 8‐, 4‐, and 2‐fold using the assay diluent. For spike recovery experiments, the authors spiked their plasma samples with 6.7 pg/mL pT127‐tau. The authors showed that after both dilution linearity and spiking, the pT217‐tau recovered values were within the acceptable range of ± 20% for these experiments.^2^, ^3^ However, the immunoassay validations deviated from established protocols commonly used in such assays. This may affect the assay's accuracy and consistency across a wider range of concentrations.

Therefore, it is notable to mention how immunoassay validation is typically done and interpreted^2^, ^3^, ^4^ (Figure 1). Standard immunoassay validation methods ensure the accuracy and reliability of the assay across a broad range of conditions.

**FIGURE 1 alz13707-fig-0001:**
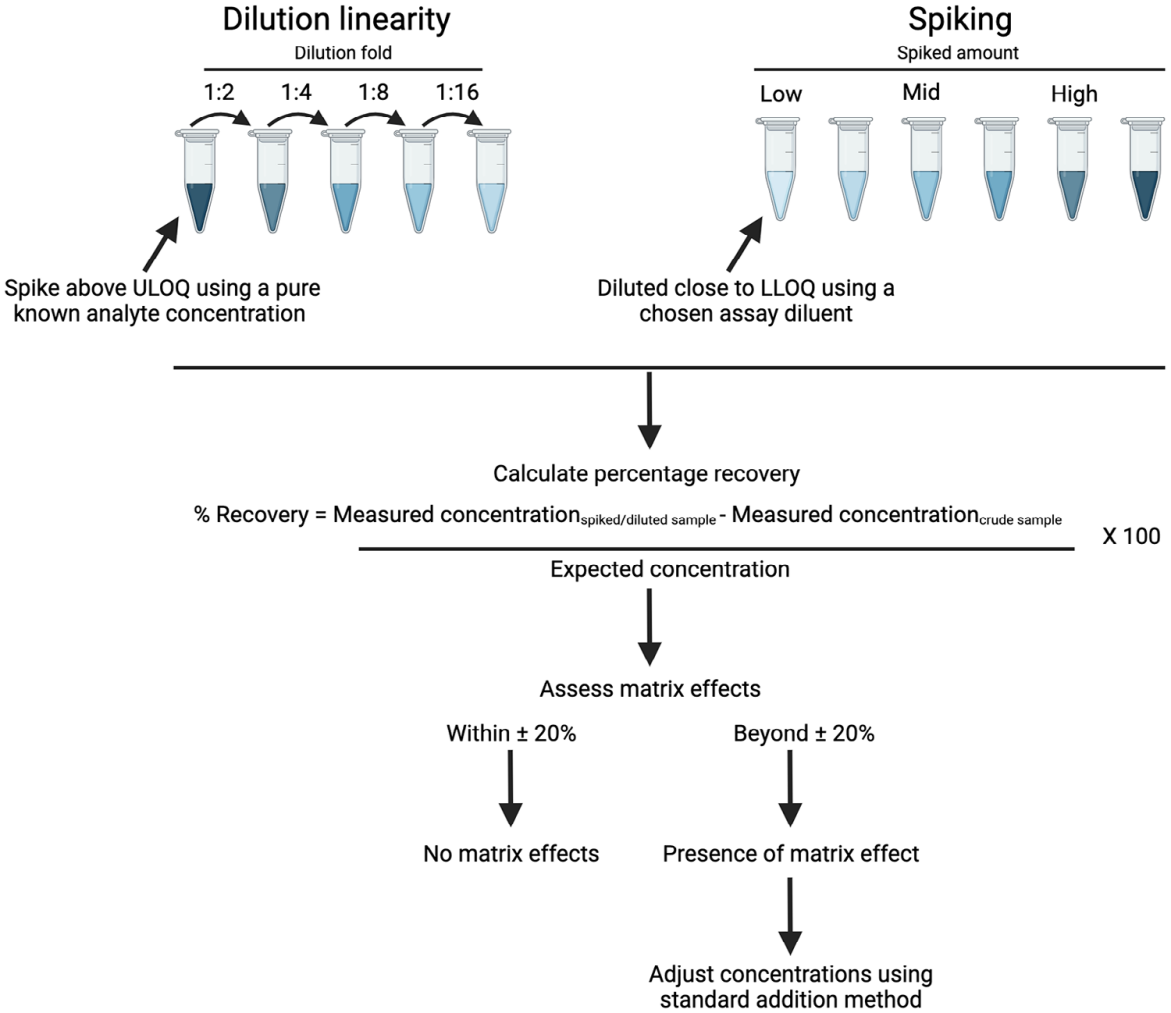
Standard procedures for analytically validating a newly developed assay. LLOQ, lower limit of quantification; ULOQ, upper limit of quantification.

Dilution linearity experiments are essential for assessing the analytical validity of an assay, particularly in understanding the interactions between the assay diluent and the medium of interest. They are done by first spiking the sample of interest with a known analyte concentration to a concentration above the upper limit of quantification (ULOQ) using, for example, the highest calibrator. Then, the samples are diluted with the assay diluent of interest using 2‐, 4‐, 8‐, and 16‐fold dilutions. The purpose here is to validate the consistency of the assay across a spectrum of diluent concentrations, a critical factor for assays dealing with samples that may exhibit a wide range of analyte levels that may be incompatible with the assay's diluent.

As for spiking experiments, which are important for determining how well the assay accurately detects the analyte at different concentrations without introducing matrix effects, the sample of interest should be first diluted slightly below or close to the lower limit of quantification (LLOQ) of the assay using the assay diluent. Then, known amounts of the analyte are added, ideally covering the entire dynamic range of the assay. For instance, in a range of 10.0 to 100.0 pg/mL, spiking could be done at various points such as 25 pg/mL (low spike), 50 pg/mL (medium spike), and 75 pg/mL (high spike). This approach validates that the assay can detect the analyte reliably across its entire dynamic range.

The recovery percentage is then calculated by comparing the measured concentration after dilution or spiking to the expected concentration, providing a clear measure of the assay's accuracy and precision at different levels. Matrix effects, caused by components in the sample that interfere with the assay, often lead to recovery rates outside of the ± 20% range, significantly compromising the assay's accuracy.

When a matrix effect is suspected, the standard addition method^5^ is typically used to address this issue by adding incremental, known amounts of the analyte to the sample matrix and then measuring the response of the assay to these additions. The true concentration of the analyte in the original sample can then be determined by extrapolating the linear response.^5^ This approach provides a more accurate picture of the bona fide analyte levels in the included samples. Other valuable information that can be easily calculated using the calibrator concentrations include signal to background (S/B), signal to noise (S/N), total error (TE), and relative error (RE),^6^ providing insights into the validity of the assay.

As for clinical utility, it is essential to test the pT217‐tau assay on *post mortem* brain tissue from persons with AD alongside controls. A crucial aspect of this testing involves correlating the results of plasma assays with brain tissue findings and comparing the diagnostic accuracy of these tests against the confirmed neuropathological diagnosis. It helps establish whether the new pT217‐tau assay outcomes are consistent with the established neuropathological diagnostic exam, thereby confirming the assay's real‐world utility.

Finally, development of in‐house assays is a time‐consuming, labor‐ and cost‐intensive process. It is important that future developers adhere to guided principles of assay development before embarking on their experiments.

## CONFLICT OF INTEREST STATEMENT

The author declares no conflict of interest. Author disclosures are available in the supporting information.

## Supporting information

Supporting Information
